# Inhibitory Effect of a French Maritime Pine Bark Extract-Based Nutritional Supplement on TNF-*α*-Induced Inflammation and Oxidative Stress in Human Coronary Artery Endothelial Cells

**DOI:** 10.1155/2015/260530

**Published:** 2015-11-17

**Authors:** Kristine C. Y. McGrath, Xiao-Hong Li, Lucinda S. McRobb, Alison K. Heather

**Affiliations:** ^1^Molecular Biosciences Team, School of Life Sciences, University of Technology Sydney, Broadway, NSW, Australia; ^2^Department of Endocrinology, Dezhou People's Hospital, Shandong, China; ^3^Department of Clinical Medicine, Macquarie University, Sydney, NSW 2109, Australia; ^4^Department of Physiology, Otago School of Medical Sciences, University of Otago, Dunedin, New Zealand

## Abstract

Oxidative stress and inflammation, leading to endothelial dysfunction, contribute to the pathogenesis of atherosclerosis. The popularity of natural product supplements has increased in recent years, especially those with purported anti-inflammatory and/or antioxidant effects. The efficacy and mechanism of many of these products are not yet well understood. In this study, we tested the antioxidant and anti-inflammatory effects of a supplement, HIPER Health Supplement (HIPER), on cytokine-induced inflammation and oxidative stress in human coronary artery endothelial cells (HCAECs). HIPER is a mixture of French maritime pine bark extract (PBE), honey, aloe vera, and papaya extract. Treatment for 24 hours with HIPER reduced TNF-*α*-induced reactive oxygen species (ROS) generation that was associated with decreased NADPH oxidase 4 and increased superoxide dismutase-1 expression. HIPER inhibited TNF-*α* induced monocyte adhesion to HCAECs that was in keeping with decreased expression of vascular cell adhesion molecule-1 and intercellular cell adhesion molecule-1 and decreased nuclear factor-kappa B (NF-*κ*B) activation. Further investigation of mechanism showed HIPER reduced TNF-*α* induced I*κ*B*α* and p38 and MEK1/2 MAP kinases phosphorylation. Our findings show that HIPER has potent inhibitory effects on HCAECs inflammatory and oxidative stress responses that may protect against endothelial dysfunction that underlies early atherosclerotic lesion formation.

## 1. Introduction

Chronic subacute inflammation and oxidative stress leading to endothelial dysfunction underlie the early pathogenesis of atherosclerosis [1, 2]. A key early step in atherosclerotic lesion formation is the adhesion of monocytes to the endothelium and the subsequent migration of the monocytes into the subintima where they engulf oxidized LDL and become classical “foam cells” [3]. The interaction of monocytes with endothelial cells is mediated by cell adhesion molecules, the most important of which are vascular cell adhesion molecule-1 (VCAM-1) and intercellular cell adhesion molecule-1 (ICAM-1) expressed by the endothelial cells [4]. The expression of both VCAM-1 and ICAM-1 is regulated by nuclear factor-kappa B (NF-*κ*B), a transcription factor that is activated by oxidative stress.

Inflammation and oxidative stress are considered key targets to improving or reversing endothelial dysfunction associated with early atherosclerotic plaque formation [1, 2]. In addition to main stream medical treatments, there is increasing popularity in using natural compounds for their intrinsic anti-inflammatory and antioxidant properties. HIPER Health Supplement (HIPER) was formulated using a combination of French maritime pine bark extract (*Pinus pinaster*, PBE), aloe vera, honey, and papain. For PBE, aloe vera, and honey, there is existing scientific literature supporting either anti-inflammatory or antioxidant properties [5–9]. In this study, we have investigated whether HIPER, a combination of these ingredients, could protect human coronary artery endothelial cells (HCAECs) from cytokine-induced inflammatory and oxidative stress responses. Our findings show that HIPER was effective in suppressing VCAM-1 and ICAM-1 expression, primarily via its effect on decreasing NF-*κ*B and MAP kinase activation. This anti-inflammatory effect is most likely due to the effect of HIPER on lowering intracellular ROS levels.

## 2. Materials and Methods

### 2.1. HIPER Health Supplement

HIPER Health Supplement, as well as the individual components of aloe vera, honey, papain, and PBE, was kindly donated by Plasmaide Pty Ltd. (Sydney, NSW, Australia). The supplement was filtered through a 0.22 *μ*m pore size sterile syringe filter unit before use (Merck Millipore, Bayswater, VIC, Australia). HIPER was used at a volume of 25 *μ*L or 50 *μ*L per mL of media that equated to a 50 mL or 100 mL dose recommended for human consumption.

### 2.2. Cell Culture

HCAECs (Cell Applications, San Diego, CA, USA) were cultured in MesoEndo Cell Growth Medium (Cell Applications). HCAECs were pretreated with HIPER for 3 h at a dose of 6.25, 12.5, 25, or 50 *μ*L per mL of media or phosphate-buffered saline (PBS; Astral Scientific, Sydney, NSW, Australia) as vehicle-control and then stimulated with TNF-*α* (1–5 ng/mL) (Sigma-Aldrich, Castle Hill, NSW, Australia) for 1 h.

### 2.3. RT-qPCR

Total RNA was extracted using TRI reagent (Sigma-Aldrich) and the concentration was normalized to 100 ng/*μ*L using a NanoDrop 1000 spectrophotometer (Thermo Fisher Scientific, Mulgrave, VIC, Australia). cDNA was generated from 100 ng of total RNA using iSCRIPT (Bio-Rad, Regents Park, NSW, Australia). An aliquot of each cDNA sample (1 *μ*L) was amplified by qPCR in reaction mixtures containing primers (12 pmol each) and iQ SYBR Green Supermix (Bio-Rad). Sequences of the primers used in the qPCR reaction were as follows: human NOX4 forward: 5′-GTGGTGGTGCTATTCCTCAT-3′, reverse: 5′-GCTGGTTCGGTTAAGACTGA-3′; human SOD-1 forward: 5′-GCGAGTTATGGCGACGAA-3′, reverse: 5′-CAGTCAGTCCTTTAATGCTTCC-3′; human VCAM-1 forward: 5′-ATGTAGTGTCATGGGCTGTG-3′, reverse: 5′-GGAATGAGTAGAGCTCCACC-3′; human ICAM-1 forward: 5′-CCATCTACAGCTTTCCGGCGC-3′, reverse: 5′-CTCTGGGGTGGCCTTCAGCA-3′; human *β*2-microglobulin (B_2_M) forward: 5′-CATCCAGCGTACTCCAAAGA, reverse: 5′-GACAAGTCTGAATGCTCCAC; and human GAPDH forward: 5′-CGATGCTGGCGCTGAGTACGT-3′, reverse: 5′-CCTGCAAATGAGCCCCAGCCTTC-3′. Amplification was performed in a BioRad iQ5 thermocycler (Bio-Rad) using the following protocol: 95°C for 30 sec, Tm of specific primer sets for 30 sec, and 72°C for 30 sec. Relative changes in mRNA levels were determined by the ΔΔC_T_ method [10], using human B_2_M and GAPDH as the reference genes.

### 2.4. Monocyte Adhesion Assay

HCAECs were pretreated with HIPER (25 *μ*L/mL) for 3 h before treated with TNF-*α* (1 ng/mL) for a further 3 h. After treatment, monocyte to endothelial cell adhesion assays were performed as previously described [11].

### 2.5. Enzyme-Linked Immunosorbent Assay (ELISA)

HCAECs were plated in 96-well plates and pretreated with HIPER (25 *μ*L/mL) and for the MAP kinase experiments, either p38 MAP kinase inhibitor SB203580 (Calbiochem/EMD Chemicals Inc., Gibbstown, NJ, USA) or MEK1/2 inhibitor UO126 (Calbiochem/EMD Chemicals Inc.) for 3 h. All cells were then treated with TNF-*α* (1 ng/mL) for a further 3 h. After treatment, ELISA was performed as previously described for VCAM-1 and ICAM-1 [11]. I*κ*B*α* levels were measured by FunctionELISA I*κ*B*α* (Active Motif, Carlsbad, CA, USA). p38 MAP kinase levels were measured by human/mouse phospho-p38 MAP kinase (T180/Y182) immunoassay (R&D Systems Inc., Minneapolis, MN, USA). MEK1/2 levels were measured by the commercially available FACE MEK1/2 ELISA kit (Active Motif).

### 2.6. NF-*κ*B Nuclear Translocation Assay

HCAECs were exposed to HIPER (25 *μ*L/mL) for 3 h and then stimulated with TNF-*α* (1 ng/mL) for 3 h. After treatment, nuclear proteins were extracted using the NucBuster protein extraction kit (Merck Millipore) and nuclear NF-*κ*B levels were then determined as previously described [12].

### 2.7. Measurement of Intracellular Reactive Oxygen Species (ROS)

Intracellular ROS levels were measured using the DCF assay (Thermo Fisher Scientific). HCAECs were pretreated with HIPER (25 or 50 *μ*L/mL) for 3 h before stimulation with TNF-*α* (5 ng/mL) for a further 3 h. After treatment, media were removed and cells washed with 1x PBS. H_2_DCFDA stain (Thermo Fisher Scientific) was diluted in 1x PBS to a concentration of 10 *μ*M before treatment of cells. The cells were incubated at 37°C for 12 min before washing with 1x PBS and ROS level was determined by fluorescence measurement (485 nm/535 nm).

### 2.8. Statistical Analysis

All data are expressed as mean ± SEM. Differences between conditions were determined by one-way ANOVA with Bonferroni's post hoc test analysis (GraphPad PRISM Software Version 4.03). Significance was set at *P* < 0.05.

## 3. Results

### 3.1. HIPER Suppressed TNF-*α*-Induced ROS Levels in HCAECs

**Figure 1:**
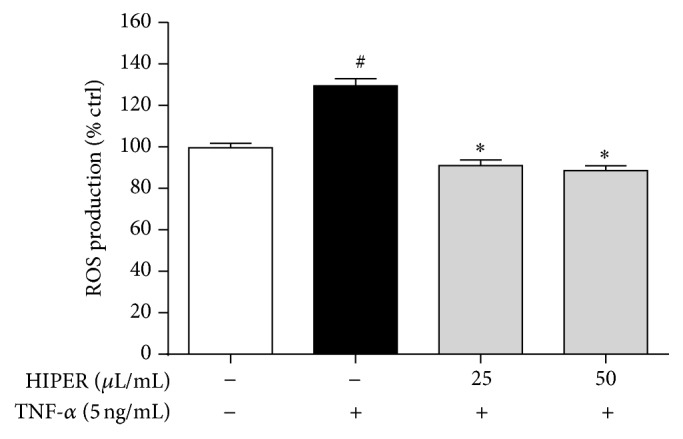
HIPER reduced ROS levels in TNF-*α*-activated HCAECs. HCAECs were treated with HIPER at doses of 25 or 50 *μ*L/mL for 3 h before activation with 5 ng/mL TNF-*α* for 3 h. ROS levels were measured using the DCF assay. Data are shown as mean ± SEM (*n* = 3). ^#^ *P* < 0.05 versus control, ^*∗*^ *P* < 0.05 versus TNF-*α*.

TNF-*α* treatment of HCAECs increased ROS levels (Figure 1). Pretreatment with HIPER abrogated the TNF-*α* effect in a dose-dependent manner (*P* < 0.05).

### 3.2. HIPER Modulated the Expression of Enzymes Involved in the Oxidative Stress Response

**Figure 2:**
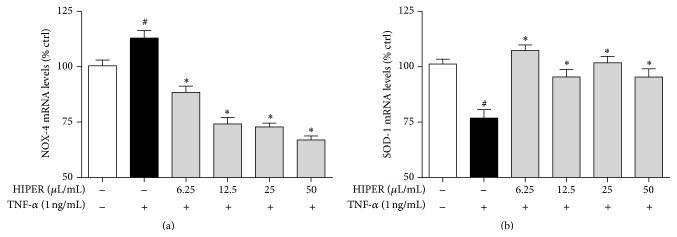
HIPER modulated NOX4 and SOD1 mRNA levels in TNF-*α*-activated HCAECs. HCAECs were treated with HIPER at concentrations of 6.25, 12.5, 25, and 50 *μ*L/mL for 3 h, before activation with 1 ng/mL TNF-*α* for 1 h. Total RNA was extracted and NOX4 (a) and SOD-1 (b) mRNA levels were measured by RT-qPCR. Data are shown as mean ± SEM (*n* = 3). ^#^ *P* < 0.05 versus control, ^*∗*^ *P* < 0.05 versus TNF-*α*.

TNF-*α* treatment increased NADPH oxidase 4 (NOX4) expression by 15%, a result that was abrogated in HCAECs pretreated with 25 or 50 *μ*L/mL HIPER for 3 h (Figure 2(a); *P* < 0.05). In contrast, TNF-*α* decreased superoxide dismutase-1 (SOD-1) expression by 24% (Figure 2(b); *P* < 0.05), which was also abrogated by HIPER pretreatment at both the 25 and 50 *μ*L/mL concentrations (*P* < 0.05).

### 3.3. HIPER Suppressed TNF-*α*-Induced VCAM-1 and ICAM-1 Expression and Monocyte Adhesion to HCAECs

**Figure 3:**
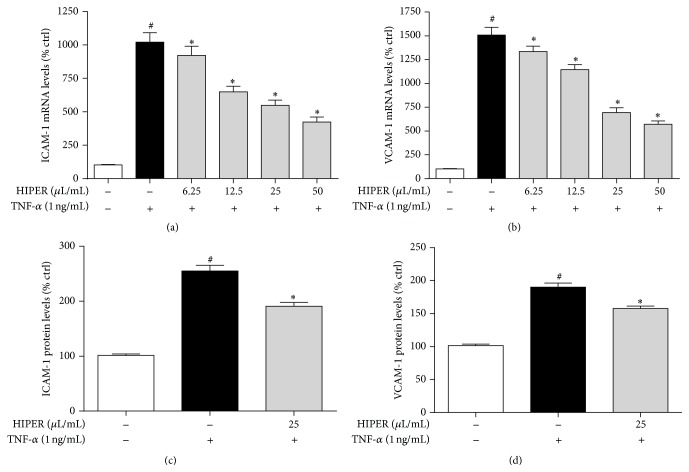
HIPER reduced ICAM-1 and VCAM-1 mRNA and protein levels in TNF-*α*-activated HCAECs. HCAECs were treated with HIPER at doses of 6.25, 12.5, 25, and 50 *μ*L/mL for 3 h, before activation with TNF-*α* (1 ng/mL) for 1 or 3 h for mRNA or protein levels, respectively. Total RNA was extracted and ICAM-1 (a) or VCAM-1 (b) mRNA levels were measured by RT-qPCR. Cell-based ELISA was used to measure ICAM-1 (c) and VCAM-1 (d) protein levels. Data are shown as mean ± SEM (*n* = 3). ^#^ *P* < 0.05 versus control, ^*∗*^ *P* < 0.05 versus TNF-*α*.

HCAECs were pretreated with HIPER (25 *μ*L/mL) and then stimulated with TNF-*α*. Figures 3(a) and 3(b) show that there was a dose-dependent decrease in ICAM-1 (a) and VCAM-1 (b) mRNA levels in response to HIPER. The decrease in mRNA levels correlated with a decrease in protein as measured by ELISA where HIPER (25 *μ*L/mL) reduced ICAM-1 protein levels by 25% (Figure 3(c); *P* < 0.05) and VCAM-1 protein levels by 18% (Figure 3(d); *P* < 0.5).

**Figure 4:**
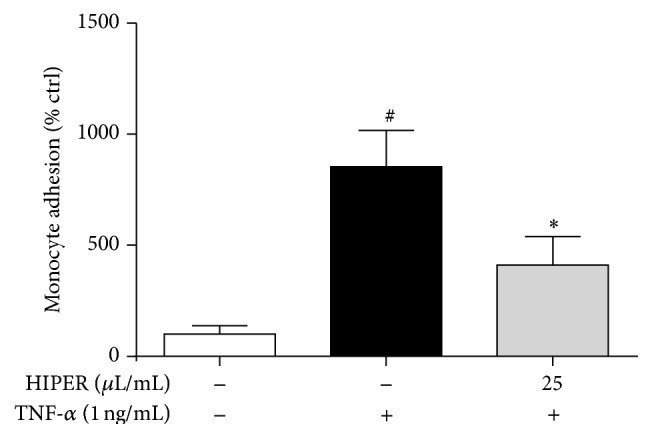
HIPER reduced monocyte adhesion to TNF-*α*-activated HCAECs. HCAECs were treated with HIPER at a dose of 25 *μ*L/mL for 3 h, before activation with TNF-*α* (1 ng/mL) for 3 h. HCAECs were then exposed to monocytes (1 × 10^6^ cells/mL) for 1 h after which nonadhered monocytes were removed and the percentage of adherent monocytes calculated. Data are shown as mean ± SEM (*n* = 3). ^#^ *P* < 0.005 versus control, ^*∗*^ *P* < 0.05 versus TNF-*α*.

Figure 4 shows that TNF-*α* stimulated monocyte adhesion to HCAECs by 8.5-fold (*P* < 0.05). Pretreatment of HCAECs with HIPER (25 *μ*L/mL) suppressed TNF-*α*-stimulated monocyte adhesion by 2-fold (*P* < 0.05).

### 3.4. HIPER Suppressed TNF-*α*-Stimulated NF-*κ*B Activation

**Figure 5:**
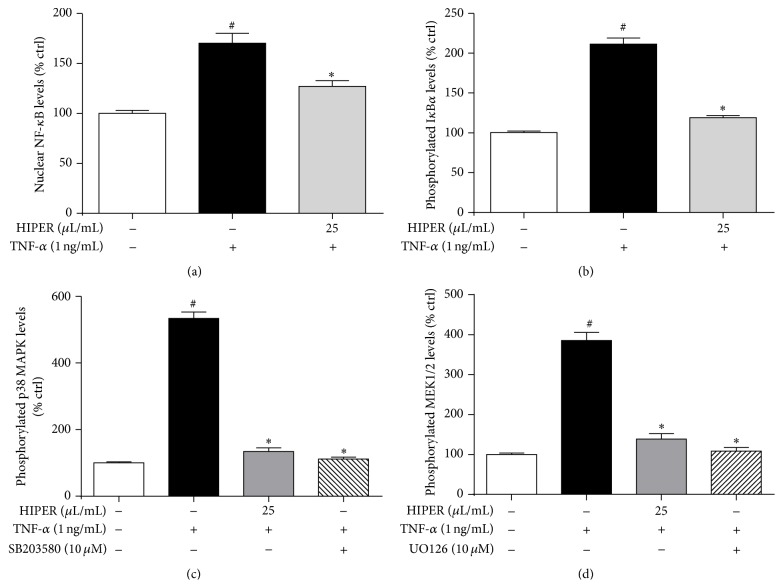
HIPER reduced NF-*κ*B activation in TNF-*α*-activated HCAECs. HCAECs were treated with HIPER at a dose of 25 *μ*L/mL for 3 h, before activation with 1 ng/mL TNF-*α* for 3 h. For (c) additional cells were treated with SB203580 (10 *μ*M) and (d) UO126 (10 *μ*M). For (a) nuclear extract was obtained and NF-*κ*B levels were measured using commercially available NoShift assay. For (b)–(d), cultured cells were used directly in commercially available ELISA kits. Data are shown as mean ± SEM (*n* = 3) ^*∗*^ *P* < 0.05 versus control, ^#^ *P* < 0.05 versus TNF-*α*.

Given that HIPER suppressed TNF-*α*-induced ROS levels and VCAM-1 and ICAM-1 expression, it was next explored whether HIPER suppressed TNF-*α*-induced NF-*κ*B activation. In its inactivated state, NF-*κ*B is bound by an inhibitor protein, I*κ*B*α*. In inflammatory conditions, cell signalling cascades that involve MAP kinases lead to the phosphorylation of I*κ*B*α* [13]. Phosphorylated I*κ*B*α* is targeted for degradation freeing NF-*κ*B to translocate to the nucleus to active target gene expression that includes VCAM-1 and ICAM-1.  Figure 5(a) shows that TNF-*α* significantly increased NF-*κ*B nuclear activation by 70% (*P* < 0.05) and that pretreatment of HCAECs with HIPER (25 *μ*L/mL) decreased this effect by 43% (*P* < 0.05).  Figure 5(b) shows that the effect of HIPER on suppressing NF-*κ*B activation was associated with a decrease in phosphorylated I*κ*B*α*, the inhibitor protein of NF-*κ*B. Phosphorylation of I*κ*B*α* is driven by MAP kinase activation. Figures 5(c) and 5(d) show that HIPER suppressed p38 and MEK1/2 MAP kinases activation almost to the same basal level as the p38 inhibitor SB203580 or the MEK1/2 inhibitor, UO126. Together, this data suggests that HIPER suppresses the HCAECs inflammatory response, at least in part, via suppression of MAP kinase and NF-*κ*B activation.

### 3.5. PBE Is Central to the Anti-Inflammatory Effect of HIPER

**Figure 6:**
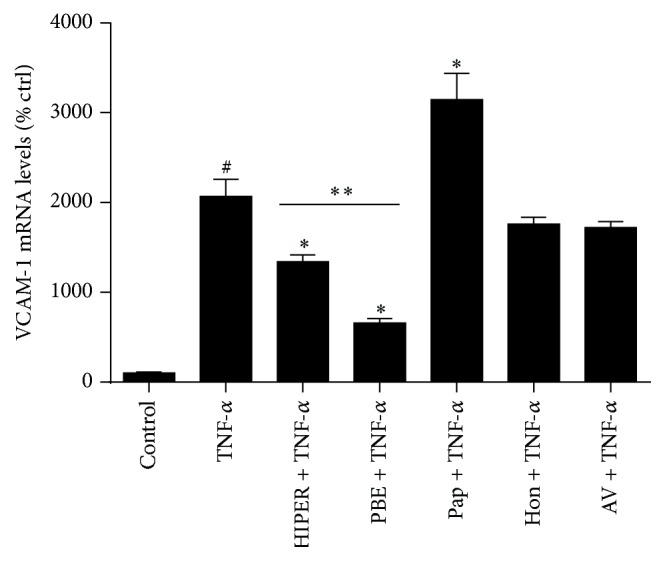
Anti-inflammatory effects of individual HIPER components. HCAECs were treated with HIPER or pine bark extract (PBE), papain (Pap), honey (Hon), or aloe vera (AV) for 3 h and then activated with 1 ng/mL TNF-*α* for 1 h. Total RNA was extracted and VCAM-1 mRNA levels were measured by RT-qPCR. Data are shown as mean ± SEM (*n* = 3). ^#^ *P* < 0.05 versus control, ^*∗*^ *P* < 0.05 versus TNF-*α*, and ^*∗∗*^ *P* < 0.05 HIPER versus PBE.

To test each HIPER ingredient for an individual anti-inflammatory effect, HCAECs were exposed to either PBE (130 *μ*g/mL or 260 *μ*g/mL), aloe vera (1.75 mg/mL), honey (0.1%), or papain (2.4 mg/mL) for 3 h before being activated with TNF-*α* (1 ng/mL) for a further 3 h. The anti-inflammatory effect of each individual component was compared to each other and to the parent compound, HIPER (25 *μ*L/mL).  Figure 6 shows that HIPER (25 *μ*L/mL) and PBE (at both concentrations) decreased TNF-*α*-induced VCAM-1 mRNA levels. PBE was twice as effective as HIPER in decreasing TNF-*α*-induced VCAM-1 expression (1.5-fold versus 3-fold, resp., *P* < 0.05). Honey and aloe vera showed a nonsignificant trend towards decreased VCAM-1 mRNA levels. By contrast, papain increased VCAM-1 mRNA levels (1.5-fold, *P* < 0.05) over and above expression levels induced by TNF-*α*.

## 4. Discussion

Extracts from natural substances may have the potential to complement mainstream medicine. In the present study, it was shown that HIPER Health Supplement, comprised of PBE, honey, aloe vera, and papain, decreased TNF-*α*-induced ROS levels that was associated with decreased ICAM-1 and VCAM-1 expression as well as decreased MAP kinase and NF-*κ*B activation. The physiologic consequence of the HIPER-mediated suppressed inflammatory response was decreased monocyte adhesion to HCAECs, which suggests improved endothelial cell function. Although this project was solely conducted with an* in vitro* model of cultured HCAECs, the findings suggest that HIPER may have potential to decrease key early steps in the pathogenesis of atherosclerosis, specifically the binding of monocytes to coronary artery endothelial cells.

In this study, we have shown that HIPER, a mixture of PBE, honey, aloe vera, and papain, has significant anti-inflammatory and antioxidant effects. Pretreatment of HCAECs to HIPER, at doses in keeping with the recommended daily dosage for human consumption, suppressed TNF-*α*-induced MAP kinase and NF-*κ*B activation that, in turn, decreased ICAM-1 and VCAM-1 expression, thereby decreasing monocyte adhesion. We further showed that HIPER suppressed ROS levels in TNF-*α*-activated HCAECs, possibly via modulation of key enzymes involved in superoxide generation and degradation. Potentially, this antioxidant property of HIPER may underlie the anti-inflammatory effects.

Investigation of the individual components showed that while PBE, honey, and aloe vera were anti-inflammatory, albeit to different extents, papain was proinflammatory. This is in keeping with its enzymatic role as a peptidase [14]. The use of papain in HIPER, therefore, requires further investigation as our findings suggest adverse effects that are potentially decreasing the efficacy of complete HIPER. On the other hand, PBE was shown to be very effective at attenuating VCAM-1 mRNA levels. PBE is extracted from the bark of the French maritime pine tree,* Pinus pinaster*, and is commercially available as a herbal dietary supplement (registered trade name: Pycnogenol). It is already in use for the treatment of many inflammatory, autoimmune, and cardiovascular disorders [8, 9, 15–17] and very recently it was shown to reduce atherosclerotic lesion burden in a small clinical study [18]. PBE is increasingly recognised for its anti-inflammatory and antioxidant effects* in vitro* and* in vivo *(reviewed in [9]). For example,* in vitro* studies have shown that PBE reduces cell adhesion molecule expression in human umbilical vein endothelial cells through suppression of NF-*κ*B [19], which is in keeping with our current findings for HIPER that show decreased NF-*κ*B activation and decreased CAM expression in HCAECs. Similarly,* in vitro* studies have demonstrated that PBE reduces ROS levels in renal tubular cells in association with decreased lipid peroxidation [20], again in accordance with our data for HIPER showing decreased ROS levels in HCAECs.* In vivo*, a recent randomized, double-blind, placebo-controlled crossover showed that 8 weeks of Pycnogenol treatment (200 mg/day) improves endothelial function in patients with coronary artery disease by reducing oxidative stress [21]. Therefore, it would now be prudent not only to test whether HIPER is able to recapitulate its* in vitro *effects when administered* in vivo* but also to test whether synergistic actions between PBE, honey, and aloe vera occur* in vivo*.

Limitations of our study include the facts that that it is an* in vitro* investigation and the bioavailability and/or bioactivation of HIPER and its components were not investigated. In this study HCAECs were directly treated with the HIPER to investigate the effects. However,* in vivo*, HIPER would be digested and metabolized by the liver that may generate metabolites that provide further or no benefit. Therefore, effects of direct HIPER exposure on HCAECs may differ from the effects when HCAECs are exposed to the metabolites of HIPER.

In conclusion, our results demonstrate the anti-inflammatory and antioxidant potential of HIPER containing the natural products, PBE, honey, aloe vera, and papain. Our finding that HIPER has a potent ability to decrease NF-*κ*B and MAP kinase activation, and therefore inflammatory gene expression, suggests that HIPER could be beneficial against a number of inflammatory conditions where cell adhesion molecules and monocyte adhesion are an underlying pathogenic mechanism that includes atherosclerosis. Endothelial cell dysfunction resulting in the local recruitment of leukocytes to sites of inflammatory challenge is a crucial step in the initiation of the atherosclerotic process, as well as the development of advanced atherosclerosis [22]. Therefore, it would be worth investigating further the effects of HIPER on suppressing early atherosclerotic lesion formation in an* in vivo* model.
