# Corrigendum to “Inhibitory Effect of a French Maritime Pine Bark Extract-Based Nutritional Supplement on TNF-α-Induced Inflammation and Oxidative Stress in Human Coronary Artery Endothelial Cells”

**DOI:** 10.1155/2018/5642129

**Published:** 2018-11-05

**Authors:** Kristine C. Y. McGrath, Sumudu V. S. Gangoda, Xiao-Hong Li, Lucinda S. McRobb, Alison K. Heather

**Affiliations:** ^1^Molecular Biosciences Team, School of Life Sciences, University of Technology Sydney, Broadway, NSW, Australia; ^2^Department of Biomedical Sciences, Faculty of Medicine and Health Sciences, Macquarie University, Sydney, Australia; ^3^Department of Endocrinology, Dezhou People's Hospital, Shandong, China; ^4^Department of Clinical Medicine, Macquarie University, Sydney, NSW 2109, Australia; ^5^Department of Physiology, Otago School of Medical Sciences, University of Otago, Dunedin, New Zealand

In the article titled “Inhibitory Effect of a French Maritime Pine Bark Extract-Based Nutritional Supplement on TNF-*α*-Induced Inflammation and Oxidative Stress in Human Coronary Artery Endothelial Cells” [1], Dr. Sumudu V. S. Gangoda was missing from the authors' list. Dr. Gangoda contributed to the data acquisition. The corrected authors' list is shown above.
